# PPARs in the Control of Uncoupling Proteins Gene Expression

**DOI:** 10.1155/2007/74364

**Published:** 2006-11-28

**Authors:** Francesc Villarroya, Roser Iglesias, Marta Giralt

**Affiliations:** Department of Biochemistry and Molecular Biology, University of Barcelona, Barcelona 585 08007, Spain

## Abstract

Uncoupling proteins (UCPs) are mitochondrial membrane transporters involved in the control of energy conversion in mitochondria. Experimental and genetic evidence relate dysfunctions of UCPs with metabolic syndrome and obesity. The PPAR subtypes mediate to a large extent the transcriptional regulation of the UCP genes, with a distinct relevance depending on the UCP gene and the tissue in which it is expressed. UCP1 gene is under the dual control of PPAR*γ* and PPAR*α* in relation to brown adipocyte differentiation and lipid oxidation, respectively. UCP3 gene is regulated by PPAR*α* and PPAR*δ* in the muscle, heart, and adipose tissues. UCP2 gene is also under the control of PPARs even in tissues in which it is the predominantly expressed UCP (eg, the pancreas and liver). This review summarizes the current understanding of the role of PPARs in UCPs gene expression in normal conditions and also in the context of type-2 diabetes or obesity.

## CURRENT KNOWLEDGE OF THE BIOLOGY OF MITOCHONDRIAL UNCOUPLING PROTEINS

Uncoupling proteins (UCPs) are mitochondrial transporters present in the inner mitochondrial membrane. The first member of the family, UCP1, is expressed in brown adipocytes and it confers on brown adipose tissue its thermogenic capacity. UCP1 confers to the mitochondrial inner membrane an enhanced conductivity to protons, thus resulting in the uncoupling of the respiratory chain and heat production. This action of UCP1 in brown adipose tissue constitutes the main molecular basis for nonshivering thermogenesis in rodents in response to cold exposure and diet. The thermogenic activity of brown fat is mainly regulated by norepinephrine released from the sympathetic nervous system innervating the tissue, acting through *β*-adrenergic, cAMP-dependent pathways. Accumulating pieces of evidence over more than two decades have indicated that energy expenditure processes elicited by UCP1 are involved in the control of energy balance, and that UCP1 activity in brown adipose tissue may provide the basis for diet-induced thermogenesis. In fact, obesity models in rodents are in most cases associated with low levels and activity of UCP1 in brown fat. Less clear is the role of UCP1 in human obesity, taking into account the residual amounts of brown adipocytes in adult humans. However, sensitive methodologies based on RT-PCR have revealed that remnant UCP1-expressing cells are widespread among the white adipose depots of human adults. Furthermore, genetic evidence of the association of UCP1 gene polymorphisms with disturbances of body weight in humans keeps the debate on the physiological role of UCP1 in adults ongoing [1]. The discovery in 1997 of two proteins highly similar to UCP1, named UCP2 and UCP3, with a high level of expression in humans, suggested the possibility that the role of UCP1 in the control of energy expenditure was played in humans by these two novel proteins. A decade later, the precise roles of UCP2 and UCP3 remain a matter of debate [2–4]. Like UCP1, UCP2 and UCP3 lower the mitochondrial membrane protomotive potential, but it is unclear whether dissipation of metabolic energy as heat is their primary biological function. However, their capacity to protect against obesity has been demonstrated, at least for UCP3, in experimental settings based on transgenic mice overexpressing the protein in muscle [5]. The specific involvement of UCP2 and UCP3 in the control of reactive oxygen species production or in fatty acid oxidation has been proposed. In any case, genetic approaches in humans have highlighted the involvement of both proteins in metabolic regulation and in associated disturbances such as diabetes and obesity [6].

The transcriptional control of gene expression of UCP1, UCP3, and, to a minor extent, of UCP2 determines the levels of the corresponding proteins in tissues and cells. Research in recent years has identified peroxisome proliferator-activated receptors (PPARs) as pivotal actors in the control of transcription of the UCP genes. As well as providing a basis for insight into the regulation of transcription of UCP genes in response to physiological ligands of PPARs, an understanding of the precise mechanisms and the PPAR subtypes involved in this regulation would provide the possibility of the development of pharmacological approaches to modulate the levels of UCPs, given the availability of drugs acting selectively on PPAR subtypes, such as fibrates and thiazolidinediones.

## PPARS IN THE CONTROL OF THE UCP1 GENE, BROWN ADIPOCYTE DIFFERENTIATION, AND ENERGY EXPENDITURE

### The UCP1 gene is a target of dual regulation by PPAR*γ* and PPAR*α* in brown adipose tissue

Brown adipose tissue and white adipose tissue have distinct metabolic functions. In contrast to the role of white adipose tissue as a site of energy storage, brown fat dissipates metabolic energy as heat, thus promoting energy expenditure. Whereas large amounts of white adipose tissue are associated with obesity, the development of high levels as well as high activity of brown adipose tissue is usually associated with a reduction in body weight. However, brown adipocytes and white adipocytes share multiple metabolic features and gene expression patterns, such as those related to lipid storage. They also share key transcriptional factors that mediate their differentiation process; namely, PPAR*γ* and CCAAT-enhancer binding-protein *α* (C/EBP*α*). In fact, all three PPARs are expressed in brown fat [7], and their relative roles in regulating brown fat thermogenesis and in UCP1 gene expression will be discussed.

PPAR*γ* is highly expressed both in brown and white adipocytes. Activation of PPAR*γ* induces brown and white adipocytes differentiation by regulating the expression of genes involved in adipogenesis and lipid storage, whereas PPAR*γ*-null cells cannot differentiate into adipocytes [8]. Mice that specifically lack PPAR*γ* in adipose tissues have reduced adiposity and compromised survival of mature brown and white adipocytes [9, 10]. Furthermore, the transcription factor C/EBP*α*, which is necessary for white adipose tissue development in mice [11], also has a critical role in brown adipocyte differentiation during perinatal development, although later on C/EBP*β* and C/EBP*δ* can functionally replace C/EBP*α* [12]. C/EBP*α* (and also C/EBP*β* and C/EBP*δ*) function synergistically with PPAR*γ* to regulate genes expressed in both brown and white adipocytes [13], but also the brown fat-specific UCP1 gene [14–16]. In fact, the transcription of the UCP1 gene is tightly regulated during brown adipocyte differentiation and in response to thermogenic activation. The 5′-flanking regions of the rat, mouse, and human UCP1 genes share a common genomic structure: a proximal regulatory region and an upstream enhancer located at −2 kb for review, see [17]. The proximal regulatory promoter contains C/EBP-regulated sites and the main cAMP-regulatory element [14, 18, 19]. The UCP1 gene distal enhancer includes a complex organization of nuclear receptor binding sites which mediate the transcriptional activation of the UCP1 gene by retinoids, thyroid hormones, PPAR agonists, and also cAMP, probably through induction of the PPAR coactivator-1*α* (PGC-1*α*) [18, 20–25].

PGC-1*α* was first identified as a PPAR*γ*-interacting protein displaying preferential expression in mature brown adipocytes rather than white adipocytes [26]. The expression of PGC-1*α* is highly induced in brown fat in response to thermogenic activation via cAMP-signaling pathways [15, 26]. PGC-1*α* has been proposed to be essential for brown adipocyte differentiation and induction of the UCP1 gene [26]. As previously mentioned, UCP1 is uniquely present in brown adipocytes, where it is highly expressed as it may account for up to 8% of the mitochondrial protein (and mitochondrial protein represents 50% of total protein). Brown adipocytes, unlike white adipocytes, also possess powerful fatty acid oxidation machinery as evidenced by the abundance of mitochondria, a high level of expression of PPAR*α* and a high activity of fatty acid oxidation pathways. PGC-1*α* can activate all of these key components of the thermogenic program through coactivation of PPAR*γ* and PPAR*α* (see below), or of transcription factors such as nuclear respiratory factor-1 [24, 26, 27]. In this way, forced expression of PGC-1*α* in white adipocytes induces mitochondrial biogenesis and expression of UCP1 [26–28]. In contrast, PGC-1*β*, another coactivator highly similar to PGC-1*α*, is only involved in controlling mitochondrial biogenesis together with PGC-1*α* [29]. Furthermore, loss of PGC-1*α* does not alter “in vitro” brown adipocyte differentiation but completely blunts the thermogenic induction via cAMP of the UCP1 gene and other thermogenic and mitochondrial genes [29].

Thiazolidinediones, drugs specifically activating PPAR*γ*, have an overall effect of promoting adipogenesis, but have also been reported to induce mitochondrial biogenesis [30] besides their direct effect upon UCP1 transcription via PPAR*γ* activation (see above). This induction of “brown fat-like” features by thiazolidinediones entails direct upregulation of transcription of the PGC1*α* gene by PPAR*γ* in adipocytes [31]. This induction of PGC1*α* is amplified by an autoregulatory loop mediated by the coactivation of PPAR*γ* action on PGC1*α* gene transcription by PGC1*α* itself [31], similarly to PGC1*α* coactivation with PPAR*γ* in the promoters of other genes such as UCP1 [24].

In summary, the available data point to a function of PGC1*α* in orchestrating the regulation of mitochondrial biogenesis and UCP1 gene induction during brown adipocyte differentiation. Regarding UCP1 gene transcription, coactivation with PPAR*γ* is probably involved in mediating this effect of PGC-1*α*. However, the thermogenic activation of mature brown adipocytes results in a negative regulation of PPAR*γ*, thus suggesting that PPAR*γ* may not be essential for UCP1 gene expression in already differentiated brown adipocytes recently reviewed in [32].

Since PPAR*α* is preferentially expressed in brown adipocytes as compared to white adipocytes, it can be expected that it is mainly through PPAR*α* that the UCP1 gene is induced in mature brown adipocytes. Agonists of either PPAR*γ* or PPAR*α* can induce UCP1 gene expression both in brown fat “in vivo” and in brown adipocytes “in vitro” [24, 33, 34]. Furthermore, the PPAR-response element of the UCP1 gene enhancer can bind either PPAR*γ* or PPAR*α* [24]. PGC-1*α* also coactivates PPAR*α*-dependent regulation of the UCP1 gene [24]. Although basal expression of UCP1 mRNA in brown fat from PPAR*α*-null mice is not altered [35], there is an impaired activation of UCP1 gene expression in PPAR*α*-null mice in several physiological situations associated with cold stress (our unpublished observations). Furthermore, genetic analyses revealed that PPAR*α* gene expression is associated with UCP1 gene induction [36].

Likewise, PGC1*α* can cooperate with PPAR*α* in the transcriptional control of genes for fatty acid catabolism in brown fat. Activation of brown fat thermogenesis, which is mediated by cAMP-dependent pathways, rapidly induces lipolysis of the stored triglycerides. Released fatty acids, in addition to being the major substrate for thermogenesis and the inducers of UCP1 uncoupling activity through direct interaction with the UCP1 protein in the inner mitochondrial membrane [37], may also act as PPAR-activators. Thus, the PGC-1*α*/PPAR*α* interaction can coordinately regulate gene expression required for active thermogenesis, including fatty acid oxidation, in mature brown adipocytes.

Whether PPAR*δ*, the third PPAR subtype, can also play a direct role in the regulation of UCP1 gene expression has not been clearly elucidated. Transgenic mice overexpressing an active form of PPAR*δ* in adipose tissues displayed reduced accumulation of triglycerides both in white fat and brown fat [38]. However, only the size of white depots was reduced. UCP1 and genes involved in fatty acid catabolism were moderately induced in brown fat and highly induced in white fat in these mice. However, neither induction of the endogenous UCP1 gene in primary murine brown adipocytes by the PPAR*δ*-specific GW501516 ligand nor PPAR*δ*-dependent regulation of the UCP1 gene promoter has been observed in brown adipocytes in culture (our unpublished observations).

### Rexinoid-dependent UCP1 gene regulation in brown adipose tissue

Both white and brown adipose tissues contain retinoic acid receptor (RAR) and retinoid X receptor (RXR) subtypes with distinct relative abundances. Retinoic- and rexinoid-dependent pathways of regulation in adipose tissues have previously been extensively reviewed [39, 40].

Retinoic acid acting via RAR has long been recognized as a potent inhibitor of the differentiation of preadipocytes into white and brown adipocytes [41, 42]. However, when retinoic acid acts upon already differentiated brown adipocytes, it dramatically increases UCP1 gene expression through a direct transcriptional effect (see below) [21]. The action of retinoic acid in promoting UCP1 gene expression has been confirmed “in vivo” by pharmacological treatment and by vitamin A supplementation of the diet [43, 44]. However, the biological significance of this powerful retinoic acid-dependent regulation of the UCP1 gene in response to RAR activation remains unknown.

Retinoic acid stimulates UCP1 gene transcription through a complex “retinoid-responsive region” in the distal enhancers of the rat or human UCP1 genes [21, 23]. Both RAR- and RXR-binding sites in the enhancer contribute to the retinoic acid effects [45]. Induction of UCP1 gene expression by retinoic acid does not require PGC1*α* [29]. The UCP1 gene is a direct target of specific RXR activators through RXR-containing heterodimers that bind to the enhancer region of the UCP1 gene [45]. Phytanic acid (3, 7, 11, 15-tetramethylhexadecanoic acid), which is a derivative of the phytol side chain of chlorophyll, has been reported to be a natural ligand of RXR subtypes [46], but also to be a direct activator of PPAR*α* [47]. Phytanic acid induces UCP1 gene expression through the RXR-binding sites in the UCP1 gene enhancer [48].This may be closely related to thermogenic activation, as phytanic acid accumulates in the brown adipose tissue fat stores and is released as a free acid when lipolysis is active in the tissue owing to thermogenic stimuli. In these conditions, phytanic acid can act as a signaling molecule linking lipolysis with enhanced synthesis of UCP1 protein to favor thermogenesis [49].

**Figure 1:**
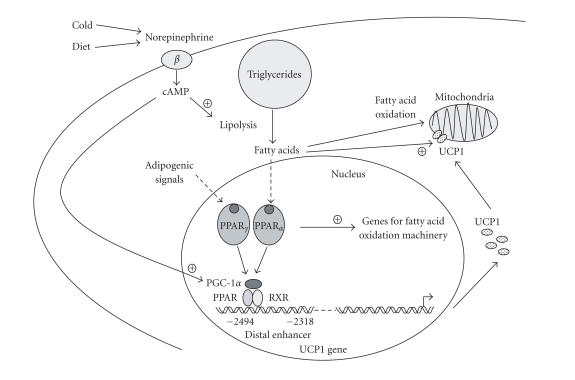
Schematic representation of the regulation of UCP1 gene expression by ligand-dependent activation of PPAR*α* and PPAR*γ*, and coactivation by PGC-1*α*. The diagram shows the PPAR response element in the rat UCP1 gene enhancer (24). Major features of the transcriptional regulation of the mouse and human UCP1 genes appear to be similar (16, 23). During brown adipocyte differentiation, adipogenic signals activate transcription of the UCP1 gene through PPAR*γ* and coactivation by PGC-1*α*, in concert with overall induction of adipocyte differentiation towards the brown fat lineage. In response to thermogenic simuli on mature brown adipocytes, activation of PPAR*α* by lipolysis-derived fatty acids contributes to the coordination of UCP1 gene transcription (thermogenesis) with the lipid oxidation pathways providing metabolic fuel for oxidation.

In summary, as depicted in Figure 1, the expression of the UCP1 gene is directly regulated by PPARs in association with adipogenic differentiation (via PPAR*γ*) and in coordination with induction of gene expression for the fatty acid oxidation required for active thermogenesis (via PPAR*α*). Whether these PPAR/rexinoid-dependent pathways can affect energy expenditure in adult humans remains to be determined. Although the amounts of UCP1-expressing brown adipocytes are low in adult humans, UCP1 gene expression can be reactivated in several conditions such as high exposure to catecholamines released by pheochromocytomas [50], or chronic treatment with antiretroviral drugs [51]. Future research will be required to determine whether PPAR agonists and/or retinoids cause similar activation, considering that they are powerful activators of human UCP1 gene transcription “in vitro” [23].

## PPAR*α* AND PPAR*δ* CONTROL UCP3 GENE EXPRESSION IN SKELETAL MUSCLE AND HEART

### Free fatty acids are major inducers of UCP3 gene expression in skeletal muscle and heart

Initial studies on the regulation of UCP3 gene expression in skeletal muscle, its main site of expression, revealed that transcript levels of UCP3 were dramatically influenced by the availability of free fatty acids to the tissue both in rodents and humans. This explained the rise in UCP3 mRNA in muscle after starvation, an observation initially considered as a paradox at the time when UCP3 was expected to have a role similar to UCP1 in the promotion of energy expenditure [52]. Today, we know that UCP3 mRNA levels are systematically upregulated in association with any physiological or experimental rise in circulating free fatty acids, either when they originate from lipolysis in white fat (starvation or exercise) or from the diet (high-fat diet) [53–55]. The increase in free fatty acids due to the initiation of milk (a fat-rich diet) intake also causes a dramatic rise in UCP3 mRNA after birth [56]. The opposite situation also occurs: a drop in free fatty acid levels such as that occurring in lactating dams is associated with a decrease in UCP3 transcript in muscle [57]. Studies in humans confirmed the regulation of UCP3 mRNA expression by fatty acids in human skeletal muscle and the heart [58, 59].

Several studies have indicated that favoring the intracellular presence of free fatty acids stimulates UCP3 gene expression. Thus, overexpression of lipoprotein lipase in muscle leads to a rise in UCP3 mRNA, surely due to the enhancement in local free fatty acid availability via hydrolysis of triglycerides [60]. Moreover, when intracellular fatty acid oxidation is blocked by the use of etomoxir, an inhibitor of carnitine palmitoyl transferase-1, UCP3 transcript levels rise also [61].

### PPAR*α* and PPAR*δ*, mediators of the fatty acid-dependent control of UCP3 transcription in skeletal muscle and heart

Multiple lines of evidence have shown that PPAR*α* plays a major role in the induction of the UCP3 gene in response to fatty acids. Acute treatment of mice pups with the specific activator of PPAR*α* Wy 14643 mimics the postnatal skeletal muscle UCP3 gene induction caused by fatty acids coming from milk [56]. A single injection of this drug to adult lactating mice also induces UCP3 mRNA expression [57]. Moreover, PPAR*α*-null mice show reduced levels of UCP3 gene expression and impaired response to starvation in the heart [62–64]. This does not occur in skeletal muscle in adult PPAR*α*-null mice, possibly due to compensatory upregulation of the UCP3 gene by PPAR*δ* (see below). However, PPAR*α*-null mice neonates display lowered UCP3 gene expression both in skeletal muscle and in the heart [65]. On the other hand, transcriptomic analysis of muscle or heart from transgenic mice which overexpress PPAR*α* specifically in these tissues revealed that UCP3 mRNA is among the most intensely induced gene transcripts [66, 67]. This occurs in concert with induction of many other genes involved in fatty acid oxidation. Thus, the UCP3 gene appears to be part of the cluster of PPAR*α*-regulated, fatty acid catabolism-related genes in the muscle and heart. Regardless of the information provided by experimental approaches directly addressing the issue of the biological function of UCP3, these observations strongly suggest that UCP3 function is likely to be related to fatty acid metabolism in these tissues.

Despite all these lines of evidence, reports on the effects of chronic treatment with fibrates, which are potential activators of PPAR*α* in muscle, have led to variable results; from unchanged expression of the UCP3 gene using Wy 14643 [33] to upregulation using bezafibrate [68]. The reasons for this variability in response to chronic treatment as opposed to the systematic upregulation observed in acute, single-injection treatment with fibrates are unclear. Perhaps the hypolipidemic consequences of chronic fibrate treatment, including reductions in the levels of circulating fatty acids, may counteract the direct positive effects of the drugs on the UCP3 gene.

Studies in cell culture have been also less conclusive in relation to the role of PPAR*α* in the control of UCP3 gene expression. Myogenic cells in culture express very low levels of UCP3 relative to muscle “in vivo” [69] and, when they were exposed to fibrates, PPAR*δ*-dependent activation appears to have a more powerful effect on UCP3 gene induction than does PPAR*α* activation [70, 71]. However, the significance of these observations for “in vivo” regulation of the UCP3 gene is unclear because myogenic cell lines such as C2C12 or L6 show abnormally reduced expression of PPAR*α* relative to that in skeletal muscle. Thus, a low sensitivity of the UCP3 gene (and other PPAR*α*-target genes) to PPAR*α* activators is anticipated in such cell systems [71, 72].

The capacity of PPAR*δ* to activate UCP3 in muscle and the heart has been demonstrated also using “in vivo” approaches. Similar to PPAR*α* overexpressing mouse models, overexpression of PPAR*δ* in muscle obtained via transgenic mice revealed that UCP3 is among the genes most sensitive to induction [73, 74]. Moreover, a mouse model of targeted disruption of PPAR*δ* specifically in the heart revealed a reduction in UCP3 levels [75]. The recent availability of drugs acting specifically on PPAR*δ* confirmed “in vivo” and “in vitro” the sensitivity of the UCP3 gene to activation via PPAR*δ*. Thus, chronic treatment of mice with a PPAR*δ* activator induces UCP3 gene expression in concert with other genes of lipid metabolism [76, 77]. Therefore, the dual regulation of the UCP3 gene by PPAR*α* and PPAR*δ* in muscle and heart is shared by many genes involved in fatty acid oxidation and again suggests the involvement of UCP3 in biological functions related to fatty acid catabolism.

Most of the above conclusions arising from studies on experimental animals or human volunteers have been confirmed by studies directly addressing the transcriptional control of the human and mouse UCP3 gene promoter in muscle cells. Both PPAR*α* and PPAR*δ* activate the UCP3 gene promoter and mediate transcriptional responsiveness to fatty acids and to drugs specifically activating both PPAR subtypes. This occurs due to the presence of a PPAR-responsive element in the proximal region of the UCP3 promoter [65, 78]. Moreover, RXR activators (rexinoids) activate UCP3 gene transcription via ligand-dependent activation of the RXR moiety of the PPAR*α*/RXR or PPAR*δ*/RXR heterodimers binding to the promoter. Interestingly, PPAR-dependent activation of the UCP3 gene requires MyoD, which acts as a transcription factor permissive for basal and PPAR-dependent regulation of the UCP3 gene in muscle cells. Coactivators such as p300 mediate this functional relationship between MyoD and PPAR-dependent regulation of the UCP3 gene [78].

The control of UCP3 gene transcription by PPAR/RXR heterodimers, which retain the capacity for ligand-dependent activation of the RXR moiety [78], explains the sensitivity of UCP3 gene expression to 9-cis retinoic acid in myogenic cells [69] and to dietary vitamin A supplementation or acute retinoic acid-treatment [79]. However, it should be taken into account that RAR-dependent pathways of regulation are also active on the UCP3 gene promoter [69]. On the other hand, although RXR has been proposed to be able to mediate transcriptional regulation through binding itself to fatty acids, UCP3 gene promoter studies appeared to exclude the possibility that RXR plays this role at the UCP3 gene [65].

**Figure 2:**
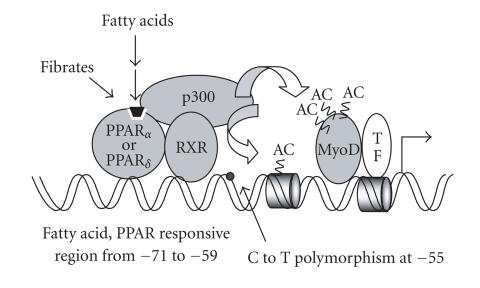
Schematic representation of the regulation of UCP3 gene transcription by PPARs. The proximal region responsive to PPAR*α* and PPAR*δ* activation via PPAR/RXR heterodimers is shown. The −55 C to T polymorphism is adjacent to this region. MyoD and TFs indicate the binding of MyoD and of basal transcription factors, respectively, close to the site of transcription initiation. P300, the main coactivator linking ligand-dependent activation of PPARs with transcriptional activation is shown. AC indicates the acetylation sites involved in transcriptional activation.

Moreover, dozens of reports in recent years have indicated a positive association between a C to T polymorphism in the human UCP3 gene promoter and body weight disturbances or insulin resistance [80]. This C to T change has been reported to modulate the relative levels of UCP3 transcripts in muscle from Pima Indians [81]. UCP3 promoter analysis revealed that the site of this polymorphism is adjacent to the PPAR*α*/*δ*-responsive element (see Figure 2), although no direct effects on promoter activity dependent on the presence of C or T have been demonstrated to date [78].

On the other hand, the potential role of PPAR*γ* in the control of UCP3 in the muscle or heart is unclear. Contradictory results have been reported on the action of thiazolidinediones on UCP3 gene expression in myogenic cells, from inhibition [82] to stimulation [83]. Treatment with thiazolidinediones “in vivo” also led to variable effects depending on the type of thiazolidinedione or the length of treatment [33, 57, 84, 85]. Mice with a muscle-specific PPAR*γ* deletion show unaltered UCP3 gene expression [86]. In these mice, treatment with rosiglitazone or troglitazone leads to a reduction in UCP3 mRNA levels whatever the genotype, thus indicating that the effects of thiazolidinediones on the UCP3 gene are likely to be PPAR*γ*-independent [86]. This is in agreement with UCP3 gene promoter studies indicating a lack of sensitivity to PPAR*γ* at least in the context of myogenic cells [65, 78].

In summary, PPAR*α* and PPAR*δ* are major regulators of UCP3 gene expression in skeletal muscle and the heart, as they appear to mediate the powerful physiological regulation of these genes by fatty acids. The physiological role of UCP3 in relation to fatty acids is unclear. However, the available data indicate that, when the muscle or heart is challenged by an overload of fatty acids, UCP3 may act to favor fatty acid metabolism in such a way that minimizes toxicity and mitochondrial production of reactive oxygen species. Pharmacological activation of PPAR*α* and PPAR*δ* via fibrates may then favor these physiological functions in muscle. Type 2 diabetes, and ultimately obesity or metabolic syndrome, may be related to the appearance of insulin resistance in muscle as a consequence of defective handling of fatty acids. The action of PPARs on the control of UCP3 gene expression may represent a potential tool to prevent the negative effects of high exposure of muscle to fatty acids, although further research will be required to more firmly establish this possibility.

## FATTY ACIDS AND PPARS IN THE CONTROL OF UCP2 GENE EXPRESSION IN SKELETAL MUSCLE AND HEART

The expression of the UCP2 gene shares with UCP3 being stimulated by fatty acids in skeletal muscle and heart, as well as being a target of PPAR*α* and PPAR*δ*-dependent activation in these tissues. However, several evidences indicate that fatty acid-dependent activation of UCP2 gene transcription is more complex, and involves also PPAR*α* and PPAR*δ*-independent mechanisms. The relative roles of these PPAR-independent mechanisms may be different depending on the tissue in which UCP2 is expressed, and, for instance, they are especially relevant in heart or other tissues such as the liver (see below). Direct effects of PPAR*δ* activators on UCP2 mRNA expression have been demonstrated in human myotubes [87], and direct analysis of regulation of the UCP2 gene promoter in muscle cells indicated that PPAR*γ* and their ligands induce promoter activity. However, no direct binding of PPAR*γ* could be detected, thus raising the possibility of an indirect effect [88].

## PPARS IN THE CONTROL OF UCP3 AND UCP2 GENE EXPRESSION IN ADIPOSE TISSUES

As previously mentioned, UCP3 is highly expressed in brown adipose tissue and to a very minor extent in white fat, whereas UCP2 is expressed in both types of adipose tissue. As in the muscle or heart, drugs activating PPAR*α* or PPAR*δ* induce UCP3 gene expression in brown fat, both as a result of acute, single-dose treatment, and after chronic treatment [33, 34].

The high expression of PPAR*γ* in adipose tissues, in contrast with that in muscle, together with the sensitivity of the UCP3 and UCP2 genes to the PPAR*α* and PPAR*δ* subtypes raised the question of the capacity of PPAR*γ* activation to affect UCP3 and UCP2 gene expression in adipose cells. The effects of chronic treatment with rosiglitazone, a thiazolidinedione capable of activating PPAR*γ*, have been reported to involve a robust induction [89], a moderate increase [90] or even no change [33] in UCP3 mRNA levels in white adipose tissue. The reasons for these discrepancies are unclear and different doses or rodent species and strains used may be the basis of the different findings. It should be noted that, as mentioned for UCP1, any treatment of mice or cells driving the white fat phenotype into a brown fat-like phenotype or generally promoting brown fat differentiation may result in increased UCP3 gene expression in white adipose depots. This UCP3 mRNA induction in white adipose depots could be just one more symptom of the acquisition of “brown fat-like” features, considering the plasticity of adipose depots in rodents. Rosiglitazone treatment “in vivo” may exert these overall effects and its action on UCP3 gene expression may depend on the extent of alterations in the brown versus white pattern of gene expression.

Concerning UCP2, chronic thiazolidinedione treatment in rodents has also been reported to increase [33] or to not affect [90] UCP2 gene expression in white fat, whereas increased expression of UCP2 mRNA has been observed in subcutaneous adipose tissue from human patients treated with rosiglitazone [91]. A moderate induction of UCP2 mRNA has also been reported in cell cultures of white adipocytes [92]. In the context of white adipogenic cell lines, PPAR*γ* and their ligands induce UCP2 promoter activity in the absence of direct binding and via E-box elements in the proximal region of the promoter [88]. In brown adipocytes, rosiglitazone as well as activators of PPAR common to the PPAR*α* and PPAR*δ* subtypes induce UCP2 mRNA expression. However, 9-cis retinoic acid and selective activators of RXR were the most powerful in inducing UCP2 mRNA expression, most probably due to their capacity to activate the dimers of RXR with PPARs or with other permissive nuclear receptors [93].

On the other hand, adipose tissues contain large amounts of endogenous triglycerides, which are capable of resulting in the local generation of free fatty acids after lipolysis. PPAR receptors can provide a mechanism for responsiveness of UCP2 and UCP3 expression to intracellularly derived fatty acids. Thus, a cross-talk between adrenergic regulation of adipose tissue lipolysis and PPAR mechanisms of induction of gene expression of UCP2 and UCP3 may occur as mentioned above for UCP1, especially in response to noradrenergic stimulus in brown adipocytes.

## ROLE OF PPARS IN THE CONTROL OF UCP2 GENE EXPRESSION IN PANCREATIC *β*-CELLS

Studies in UCP2-null mice have revealed that UCP2 exerts substantial negative control over glucose-stimulated insulin secretion [94]. Thus, UCP2 expression may play an important role in the pathogenesis of diabetes. UCP2 expression is stimulated by high glucose and/or high free fatty acid levels both “in vivo” and “in vitro”, as well as being increased in animal models of type 2 diabetes. On the other hand, a genetic deficiency of UCP2 improves *β*-cell function in animal models as well as in “in vitro” models of glucotoxicity and lipotoxicity in *β*-cells reviewed in [95].

It has been demonstrated that exposure to fatty acids increases transcription of the UCP2 gene in human and rodent cells representative of adipocytes and myocytes (see above), as well as in pancreatic *β*-cell-derived cell lines (INS-1 cells). An enhancer region has been identified between −86 to −44 of the mouse UCP2 gene. This enhancer contains Sp1 elements, sterol regulatory element (SRE), and double E-box elements all clustered together and is responsible for basal and fatty acid-stimulated transcription. The response to fatty acids appears to be mediated by sterol regulatory element binding proteins (SREBPs) binding to the SRE [96]. This enhancer is not conserved in the human UCP2 promoter but two E-box motifs at −911 to −906 and −743 to −738 have been identified as being responsible for the SREBP activation of human UCP2 gene transcription in INS-1E cells [97]. However, despite the important pathophysiological implications, the mechanisms by which chronic exposure to fatty acids increases UCP2 expression in pancreatic *β*-cells have not been completely characterized, and in addition to SREBP proteins, PPAR receptors and the G protein-coupled receptor GPR40 could be implicated.

All PPAR subtypes are expressed in pancreatic *β*-cells [98]. Although their roles in *β*-cell function remain poorly understood, several lines of evidence suggest that PPAR*α* may be implicated in the modulation of insulin secretion: (i) fatty acids stimulate the expression of PPAR*α* and its target genes in islets [98]; (ii) clofibrate treatment or PPAR*α* overexpression in INS-1cells induce UCP2 expression, increase fatty acid oxidation, and decrease basal and glucose-stimulated insulin secretion [99]; (iii) in wild-type mice, starvation increases islet PPAR*α* and UCP2 expression, which may contribute to decreased insulin secretion, whereas fasted PPAR*α* null-mice display increased plasma insulin levels and enhanced glucose-induced insulin secretion [100]. Thus, pancreatic PPAR*α* signaling appears to be significant “in vivo” and, when PPAR*α* is activated due to elevated fatty acid levels, as in obesity, it may contribute to glucose intolerance and *β*-cell dysfunction.

Contradictory data have been reported on the effects of PPAR*γ* on UCP2 expression in *β*-cells. It has been described that overexpression of PPAR*γ* causes upregulation of UCP2 expression and suppresses glucose-stimulated insulin secretion [101]. In contrast, the increase in UCP2 expression induced by chronic exposure of pancreatic islets to palmitate is prevented by addition of rosiglitazone, and this treatment also normalizes insulin secretion [102]. No direct binding of PPAR*γ* to the enhancer in the mouse UCP2 gene has been observed. Thus, the effects on UCP2 expression may be produced by indirect mechanisms [88].

GPR40 has been recently identified as a G protein-coupled receptor selectively expressed in *β*-cells and activated by fatty acids. GPR40-null mice develop neither hyperinsulinemia nor glucose intolerance when challenged with a chronic high-fat diet. In contrast, transgenic mice overexpressing GPR40 in *β*-cells are glucose intolerant and show impaired glucose-stimulated insulin secretion. In addition, in pancreatic islets of these mice, the mRNA levels of PPAR*α*, SREBP1c, and UCP2 are increased. Thus GPR40 may play a key role in the development of diabetes and could be implicated in the upregulation of PPAR*α* signaling in insulin-resistant conditions [103].

## PPARS AND UCP GENE EXPRESSION IN THE LIVER

The liver is the organ in which expression of UCPs is the lowest, in basal conditions. Only minor expression of UCP2 can be detected in the adult liver, and it is mainly due to high expression in Kupffer cells [104]. However, in situations of metabolic stress, UCP2 expression is induced in the liver, and enhanced expression appears mainly in hepatocytes [105].

Increased UCP2 mRNA expression in the liver has been reported in response to starvation, but also in obese, leptin-deficient conditions, and in rodents treated with a high-fat diet [35, 106, 107]. However, the increase in UCP2 expression is not necessarily related to obesity and insulin resistance, as a high fish-oil diet, which does not result in significant weight gain, is more effective in increasing UCP2 levels than is a high safflower oil-based diet [108]. Thus, it has been suggested that fatty acids might be key factors determining the control of UCP2 expression in the liver, regardless of whether they are associated with high lipolysis in situations of starvation or the opposite, high fatty acid levels as in obesity. PPAR signaling is a candidate for mediation of this regulation. In fact, PPAR*α* expression increases in the liver during fasting [35] and in several models of murine obesity [106]. Chronic treatment of rodents with PPAR*α* agonists such as fenofibrate or Wy 14643 increases hepatic UCP2 mRNA expression [105–108]. UCP2 mRNA levels are also upregulated in cultured hepatocytes in response to polyunsaturated fatty acids, Wy 14643 or fenofibrate [105, 109]. However, there is some data suggesting the existence of signaling mechanisms other than through PPAR*α*. For instance, the increase in liver UCP2 expression induced by starvation is preserved in PPAR*α*-null animals [35]. It has been suggested that PPAR*δ* may contribute to the regulation of UCP2 gene expression in PPAR*α*-deficient mice [110]. Regulation via PPAR*γ* must be also considered as UCP2 is induced by the PPAR*γ* activator troglitazone in cultured hepatocytes. However, the PPAR*α* activator Wy 14643 was a more powerful inducer of UCP2 gene expression in hepatic cells [109]. Despite the very low expression of PPAR*γ* in the liver under basal conditions, it is increased in obesity, in insulin resistance, and after a high-fat diet [106, 107]. PPAR*γ* is highly expressed in liver from PPAR*α* null-mice fed a high-fat diet, and this is associated with an induction of UCP2 gene expression [107]. Moreover, adenoviral-induced overexpression of PPAR*γ* in the liver of PPAR*α* null-mice causes a dramatic increase in UCP2 mRNA levels [107]. Thus, the available data suggests a major role for PPAR*α* in the regulation of UCP2 expression in the liver whereas, in some particular pathophysiological situations, additional pathways may be involved; mainly PPAR*δ* and PPAR*γ* as well as possibly other transcription factors.

Among UCP gene regulation in the liver, most attention has been focused in UCP2, as other UCP genes are silent in this tissue. However, it has been described that chronic fenofibrate administration to mice or rats induces “de novo” UCP3 expression in the liver [108, 111]. Recently, it has been demonstrated that the appearance of UCP3 transcripts is accompanied by the presence of the UCP3 protein in the mitochondrial fraction. In fact, genes involved in fatty acid oxidation and preferentially expressed in muscle, such as carnitine palmitoyl-transferase I-b, are also induced in the liver as a consequence of fenofibrate treatment [112]. Interestingly, although this treatment also upregulates UCP2 mRNA levels, UCP2 protein was not detectable, most likely due to the presence of an inhibitory post-translational mechanism. Thus, in the absence of UCP2 protein, the uncoupling effects detected in liver mitochondria after fenofibrate treatment are presumably attributable to UCP3 [112]. The results of chronic fenofibrate treatment stress the importance of post-translational mechanisms of regulation of UCP2 gene expression in the liver, in agreement with previous reports in other systems [113].

## CONCLUSIONS AND PERSPECTIVES

Intensive research efforts over recent decades have established that PPARs are major controllers of UCPs gene expression. Different PPAR subtypes are preferentially involved in the control of each UCP gene depending on the UCP gene or the main tissue of expression. The control of UCPs genes by PPAR subtypes either provides tissue-specific regulation of UCPs gene transcription, as seen in UCP1 control by PPAR*γ*, or regulates the responsiveness of UCPs genes to metabolic challenges, as seen in the control of the UCP3 gene by PPAR*α* and PPAR*δ* in the muscle and heart. The precise identification of mechanisms or PPAR subtypes involved in the control of UCP genes may be of utmost relevance in the foreseeable pharmacological approaches aimed at influencing metabolic disturbances involving skeletal muscle (ie, UCP3 gene control) or at modulating pancreatic insulin secretion (ie, UCP2 control in the pancreas). This research can be expected to have a high impact in the near future in relation to obesity and metabolic syndrome. Other issues poorly explored to date, as for instance the role of PPAR-dependent regulation of UCP2 gene expression in macrophages, cells expressing high levels of UCP2 [114] and highly sensitive to PPARs [115], would be important to further establish the mechanisms of PPAR action in inflammatory processes, including the chronic inflammation present in obesity. A new transgenic mouse model with a specific deletion of PPAR*γ* in macrophages has already been developed [116] which may be useful in exploring the role of PPAR*γ* in this cell type. We should expect much new data in the next years on the role of PPAR subtypes in obesity and metabolic syndrome, and on the role of disturbances in PPAR-mediated control of UCPs gene expression in these pathologies.
